# Resting energy expenditure, calorie and protein consumption in critically ill patients: a retrospective cohort study

**DOI:** 10.1186/s13054-016-1538-4

**Published:** 2016-11-10

**Authors:** Oren Zusman, Miriam Theilla, Jonathan Cohen, Ilya Kagan, Itai Bendavid, Pierre Singer

**Affiliations:** 1 Department of Internal Medicine E, Rabin Medical Center, Beilinson Hospital, Petah Tikva, Israel; 2 Department of General Intensive Care and Institute for Nutrition Research, Rabin Medical Center, Beilinson Hospital, Petah Tikva, Israel; 3 Nursing Department, Steyer School of Health Professions, Sackler School of Medicine, Tel Aviv University, Tel Aviv, Israel; 4 Sackler School of Medicine, Tel Aviv University, Tel Aviv, Israel

**Keywords:** Indirect calorimetry, Nutrition, Protein, Resting energy expenditure, Calorie consumption

## Abstract

**Background:**

Intense debate exists regarding the optimal energy and protein intake for intensive care unit (ICU) patients. However, most studies use predictive equations, demonstrated to be inaccurate to target energy intake. We sought to examine the outcome of a large cohort of ICU patients in relation to the percent of administered calories divided by resting energy expenditure (% AdCal/REE) obtained by indirect calorimetry (IC) and to protein intake.

**Methods:**

Included patients were hospitalized from 2003 to 2015 at a 16-bed ICU at a university affiliated, tertiary care hospital, and had IC measurement to assess caloric targets. Data were drawn from a computerized system and included the % AdCal/REE and protein intake and other variables. A Cox proportional hazards model for 60-day mortality was used, with the % AdCal/REE modeled to accommodate non-linearity. Length of stay (LOS) and length of ventilation (LOV) were also assessed.

**Results:**

A total of 1171 patients were included. The % AdCal/REE had a significant non-linear (*p* < 0.01) association with mortality after adjusting for other variables (*p* < 0.01). Increasing the percentage from zero to 70 % resulted in a hazard ratio (HR) of 0.98 (CI 0.97–0.99) pointing to reduced mortality, while increases above 70 % suggested an increase in mortality with a HR of 1.01 (CI 1.01–1.02). Increasing protein intake was also associated with decreased mortality (HR 0.99, CI 0.98–0.99, *p* = 0.02). An AdCal/REE >70 % was associated with an increased LOS and LOV.

**Conclusions:**

The findings of this study suggest that both underfeeding and overfeeding appear to be harmful to critically ill patients, such that achieving an Adcal/REE of 70 % had a survival advantage. A higher caloric intake may also be associated with harm in the form of increased LOS and LOV. The optimal way to define caloric goals therefore requires an exact estimate, which is ideally performed using indirect calorimetry. These findings may provide a basis for future randomized controlled trials comparing specific nutritional regimens based on indirect calorimetry measurements.

## Background

The provision of nutritional support for critically ill patients continues to be the subject of intense debate, with the central question being the optimal amount for the maximum benefit. In this regard, the results of recent studies have not resulted in greater clarity, as both benefit [1–3] and harm [4, 5] have been demonstrated when the caloric intake is increased towards measured goals, while the negative effects of underfeeding have also been described [6, 7]. Importantly, for many reasons either by design or default, many critically ill patients do not receive their full energy requirements and the proportion of calories delivered varies widely. Any possible shortfall in caloric intake is further compounded by the use of predictive equations to assess caloric goals. These are known to be less accurate and provide only an approximate snapshot of metabolic needs [8] as opposed to the actual measurement of resting energy expenditure (REE) by indirect calorimetry (IC), which may also be used to provide updated information about changing energy requirements. Although the use of indirect calorimetry is being increasingly encouraged [9–11], technical and economic considerations hinder its more widespread use [12].

The purpose of the present study was to assess the association between the percent of administered calories as a function of the measured REE and outcomes, including 60-day mortality, ICU length of stay and days of mechanical ventilation in a large cohort of critically ill patients. In addition, we assessed the effect of protein consumption on these same parameters.

## Methods

All patients admitted to the 16-bed multidisciplinary ICU of the Rabin Medical Center, a tertiary-care, university-affiliated hospital, from 2003 to 2015, who underwent IC measurements (Deltatrac II, Datex-Ohmeda, GE, USA) and who received enteral nutrition with or without supplemental total parenteral nutrition (TPN) were included in this study.

The calorimeter was calibrated with ethanol on a monthly basis and for test gases (ambient air and O_2_ 95 % and CO_2_ 5 %) prior to all measurements. Prior to testing, patients were required to be in a stable condition for at least 30 minutes, ventilated with inspired oxygen fraction (FiO_2_) <60 % and positive end-expiratory pressure (PEEP) <10 cm H_2_O, without any discernable air leak. Only stable measurements for at least 20 minutes were considered acceptable. Oxygen consumption and CO_2_ production were measured and the respiratory quotient (RQ) and REE calculated using the Weir equation [13]. Any IC measurement was considered the goal for that day, with subsequent measurements updating the target. The numbers of IC measurements or frequency were determined by the treating physician.

For the main analysis we included only patients with an ICU stay >96 hours or evaluable nutrition days, in order to reduce any possible bias caused by short stay, early mortality or the expectation that the effect of nutrition might necessitate at least this duration of exposure [2]. The count of length of stay and evaluable nutritional days started from the hour of arrival in the ICU.

Demographic data collected included age, sex, height and weight, acute physiology and chronic health evaluation (APACHE) II score, sequential organ failure assessment (SOFA) score, admission category (medical, surgical or trauma) and admission diagnosis (not mutually exclusive cardiovascular, respiratory, sepsis).

Nutritional parameters noted included route of feeding (enteral and/or parenteral), repeated REE measurements and insulin therapy (units/day) and the amount of total calories and protein administered daily until ICU death, discharge from the ICU or the start of exclusive oral feeding. Non-nutritional calories administered in the form of glucose infusions and propofol were included as administered calories. Protein intake was assessed as grams per day and as percent of requirement, with the goal being 1.3 g/kg. The practice in our unit is to target caloric intake as 100 % of REE. Administering supplemental parenteral nutrition for patients not reaching targets is decided by the treating physician. Gastro-intestinal parameters assessed included the presence of a gastric residue >150 ml aspirated via the nasogastric tube, diarrhea defined as at least three loose stools per day, constipation defined as ≥3 days with no bowel movement and vomiting. Readmissions to the ICU were discarded.

The percent of daily administered calories divided by REE (% AdCal/REE) and the mean value for the ICU hospitalization were calculated for each patient and its association with the outcome 60-day mortality was assessed. In order to mitigate the possible effect of the duration of exposure to nutrition on the results, we pre-planned sensitivity analyses, which included only measurements from day 3 onward, adjusting for total evaluable nutrition days and analyzing patients surviving >7 days. As death date is updated in our computer records by the Ministry of Health, we were able to record both in-hospital and post-hospital-discharge death. In addition, we assessed the association between the % AdCal/REE and length of stay (LOS) and length of ventilation (LOV). LOS was defined by physical discharge from the ICU, irrespective of medical status. The study was approved by the Rabin Medical Center institutional review board who waived the requirement for consent.

### Statistical analysis

Continuous normally distributed variables are presented as means ± standard deviations (SD) and compared using Student’s *t* test. Ordinal and/or non-normally distributed variables are presented as median and interquartile range (IQR) and compared using the Wilcoxon rank sum test. Normality was assessed using the Shapiro-Wilk test. Categorical variables were compared using the chi square test. Analysis of variance (ANOVA) for repeated measures was used to compare repeated total calorie recordings or REE, after verifying that the data followed model assumptions.

Initially an unadjusted Cox proportional hazards model for 60-day survival was used, with the percent AdCal/REE entered as a continuous variable. As the effect of AdCal/REE was assumed to be non-linear (with possible adverse outcomes due to underfeeding or overfeeding at a certain point), the percent was modeled as a restricted cubic spline with pre-specified knots [14]. Non-linearity was tested by ANOVA. An adjusted model with covariates selected based on univariate analysis, with covariate selection by bootstrapping was then fitted. Multicollinearity was assessed by variation inflation factors and *R* ^2^. Model validation was performed by bootstrapping. For the sensitivity analysis, the model was re-run with evaluable nutrition days as a covariate, with the percent calculated omitting the first 2 days and last, by restricting the sample to patients with LOS >7 days. We used the Kruskal-Wallis test for comparison of LOV and LOS. A *p* value <0.05 was considered significant. All statistical procedures were carried out in R with the appropriate packages [9–12, 15–18].

## Results

A total of 6994 patients were admitted to the ICU during the study period; 6536 of these patients were unique admissions. Of these, 5053 patients received enteral and/or parenteral feeding. Their median age was 58 (IQR 34) years and 57 % were male. The median LOS was 5 (IQR 10) days and the LOS for those remaining in the ICU for >96 hours (3019 patients) was 11 (IQR 13) days. The 60-day mortality in this group was 32 %.

Of the 5053 patients, 1375 had IC measurements, giving a total of 5012 measurements. There were 6 patients with incomplete background data and 204 with a short duration of ICU stay or evaluable nutrition days, so that 1171 patients were included in the final analysis. Three or more REE measurements were performed on 559 patients (48 %). The baseline demographic data for the study group (1171 patients), survivors (846 patients) and non-survivors (325 patients) are shown in Table 1.

**Table 1:** Patient characteristics

Variable	Overall	Survived	Died within 60 days	*P* value
Number	1171	846	325	
Age, years, median (IQR)	60.52 (28.9)	55.95 (30.82)	68.06 (19.66)	<0.001
Male gender, *n* (%)	747 (63.8)	471 (62.1)	276 (67.0)	0.106
Year of inclusion, median (IQR)	2010 (5)	2010 (5)	2010 (5)	0.01
Weight, kg, median (IQR)	80 (20)	80 (20)	80 (23.5)	0.9
Height, m, mean (sd)	1.70 (0.09)	1.70 (0.10)	1.69 (0.09)	0.027
BMI, kg/m^2^, mean (sd)	28.01 (7.82)	27.96 (7.28)	28.14 (9.08)	0.75
Metabolic				
Daily protein intake, g, mean (sd)	67.79 (21.93)	69.54 (22.09)	64.58 (21.28)	<0.001
Percent of requirement, mean (sd)	89.69 (32)	86.92 (32.76)	91.2 (31.62)	0.03
REE, mean (sd)	1944.10 (495.15)	1999.25 (485.37)	1842.50 (497.48)	<0.001
Harris-Benedict REE, mean (sd)	2304.50 (468.52)	2339.65 (443.56)	2239.73 (505.50)	<0.001
25 kcal/kg REE, mean (sd)	2019.42 (542.10)	2017.94 (507.44)	2022.13 (601.38)	0.9
Total daily calories received, kcal, mean (sd)	1651.08 (476.52)	1689.08 (478.68)	1581.07 (465.02)	<0.001
Daily enteral calories, kcal, mean (sd)	1287.43 (624.36)	1369.94 (608.32)	1135.41 (625.69)	<0.001
Daily parenteral calories, kcal, mean (sd)	363.65 (542.54)	319.14 (507.43)	445.66 (593.86)	<0.001
Parenteral calories >70 % of total, *n* (%)	134 (11.4)	65 (7,7)	69 (21.2)	<0.001
Administered calories/REE percent, mean (sd)	89 (30)	88 (28)	91 (33)	0.115
Daily insulin therapy, units, mean (sd)	44.36 (11.33)	44.46 (11.58)	44.16 (10.89)	0.679
Admission category				
Surgical, *n* (%)	499 (42.6)	363 (42.9)	136 (33.0)	<0.001
Trauma patient, *n* (%)	258 (22.0)	209 (27.5)	49 (11.9)	<0.001
Medical, *n* (%)	672 (57.4)	483 (57.1)	189 (58.1)	0.93
Sepsis, *n* (%)	267 (22.8)	152 (20.0)	115 (27.9)	0.003
Respiratory, *n* (%)	285 (24.3)	179 (23.6)	106 (25.7)	0.456
Cardiac, *n* (%)	242 (20.7)	121 (15.9)	121 (29.4)	<0.001
Severity				
APACHE II^a^, median (IQR)	22 (10)	20 (9)	26 (9.75)	<0.001
SOFA score, median (IQR)	8 (5)	7 (5)	9 (6)	<0.001
Vasopressors used, *n* (%)	904 (77.2)	534 (70.4)	370 (89.8)	<0.001
Vasopressor days, median (IQR)	4 (6)	4 (4)	6 (7)	
Gastrointestinal				
Diarrhea, *n* (%)	598 (51.1)	412 (54.3)	186 (45.1)	0.003
Diarrhea occurrences, median (IQR)	7 (13)	7 (14)	6 (12)	0.01
Constipation, *n* (%)	792 (67.6)	531 (70.0)	261 (63.3)	0.025
Constipation days, mean (sd)	6 (4)	7 (3)	6 (4)	
Large GRV or vomiting, *n* (%)	433 (37.0)	300 (39.5)	133 (32.3)	0.017
Large GRV or vomiting occurrences (median (IQR))	3(6)	3(6)	3 (7)	0.72
Laboratory data				
White blood cells, 10^9^/L, mean (sd)	12.05 (6.48)	12.19 (6.19)	11.79 (6.97)	0.316
Neutrophil count, 10^9^/L, mean (sd)	10.62 (6.04)	10.74 (5.82)	10.40 (6.42)	0.36
Platelets, 10^9^/L, median (IQR)	176 (119)	178 (111)	169.50 (134)	0.29
Fibrinogen, g/L, median (IQR)	484 (372)	442 (360.5)	543 (350.75)	<0.001
INR, median (IQR)	1.27 (0.39)	1.24 (0.35)	1.32 (0.49)	<0.001
Albumin, g/dl, mean (sd)	2.59 (0.79)	2.66 (0.81)	2.46 (0.73)	<0.001
Total bilirubin, mg/dl, median (IQR)	0.70 (0.75)	0.67 (0.67)	0.74 (0.96)	<0.001
CRP, mg/dl, median (IQR)	11.05 (17.35)	10.87 (17.57)	11.31 (16.83)	0.41
Creatinine, mg/dl, median (IQR)	0.95 (0.86)	0.84 (0.61)	1.25 (1.3)	<0.001

^a^Available only for 52 % of patients. *n* number of patients, *BMI* body mass index, *REE* resting energy expenditure, *APACHE* acute physiology and chronic health evaluation, *SOFA* sequential organ failure assessment, *GRV* gastric residual volume, *INR* international normalized ratio, *CRP* C-reactive protein

**Fig. 1:**
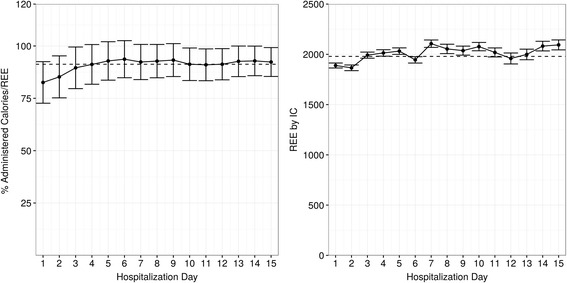
Daily mean administered calories/resting energy expenditure (*Adcal/REE*) percent by indirect calorimetry (*IC*)

The % AdCal/REE was 89 % (±30 %) for the study group, 89 % (±28 %) in patients who survived, and 91 % (±34 %) in patients who died within 60 days (*p* = 0.11). Mean REE and the % AdCal/REE by hospitalization day is shown in Fig. 1. The between-day difference was significant (*p* < 0.001 for both), but lost significance after excluding the first 2 days of hospitalization (*p* = 0.28 and *p* = 0.21 for both). The median time to first IC measurement was 35 hours from admission. The mean of the first measurement was 1901 kcal (±516) and 1980 kcal (±535) overall. The minimum REE noted was 800 kcal, while the maximum was 4540 kcal. Initiation of nutritional support was started after a mean of 4 hours from admission.

**Fig. 2:**
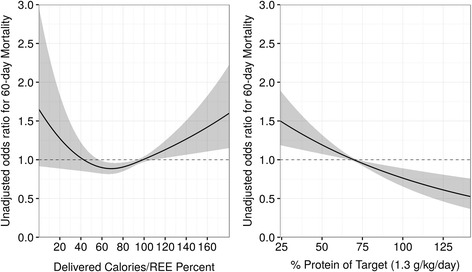
Association of administered calories/resting energy expenditure (Adcal/REE) percent with 60-day mortality (*left*), and protein intake by daily requirement (1.3 g/kg/d) with 60-day mortality (*right*) by odds ratio. *REE* resting energy expenditure

When the % AdCal/REE was examined as a continuous variable in relation to 60-day mortality, a significantly non-linear pattern was demonstrated (*p* = 0.0078) which resulted in a U-shaped curve (Fig. 2), with significant association with mortality (*p* = 0.008). The lowest mortality was noted at 70 % AdCal/REE (the minimum point of the U-shaped curve). Increasing the percent AdCal/REE from 0 % to 70 % was associated with decreasing mortality (hazard ratio (HR) 0.98, 95 % CI 0.97–0.99), while an AdCal/REE ratio ≥70 % was associated with increasing mortality (HR 1.01, 95 % CI 1.01–1.02)). An AdCal/REE >100 % was associated with a HR >1. After adjusting for other variables (shown in Table 2) the % AdCal/REE was still significantly associated with 60-day mortality (p = 0.006).

**Table 2:** Cox proportional hazards model variables for mortality by 60 days

Variable	Statistic	Std. error	Estimate	95 % CI	*P* value
Age (years)	8.94	0	1.03	1.02–1.04	<0.001
Gender (male)	2.52	0.11	1.32	1.06–1.64	0.012
Inclusion date	−2.1	0.02	0.97	0.93–0.99	0.02
Daily protein/kg	−2.37	0	0.99	0.98–0.99	0.0178
Delivered calories/REE up to 70 %	−2.93	0.01	0.98	0.97–0.99	0.006
Delivered calories/REE >70 %	3.16	0	1.01	1.01–1.02	
Surgical patient	−3.84	0.11	0.65	0.52–0.81	<0.001
Need for vasopressors	4.16	0.17	2.06	1.47–2.9	<0.001
SOFA score	5.37	0.02	1.1	1.06–1.13	<0.001
Diarrhea	−5.42	0.1	0.57	0.47–0.7	<0.001
Bilirubin total	3.21	0.02	1.05	1.02–1.09	0.001
Creatinine	3.36	0.03	1.12	1.05–1.2	<0.001
Parenteral nutrition	4.56	0.11	1.61	1.31–1.98	<0.001

*REE* resting energy expenditure

**Fig. 3:**
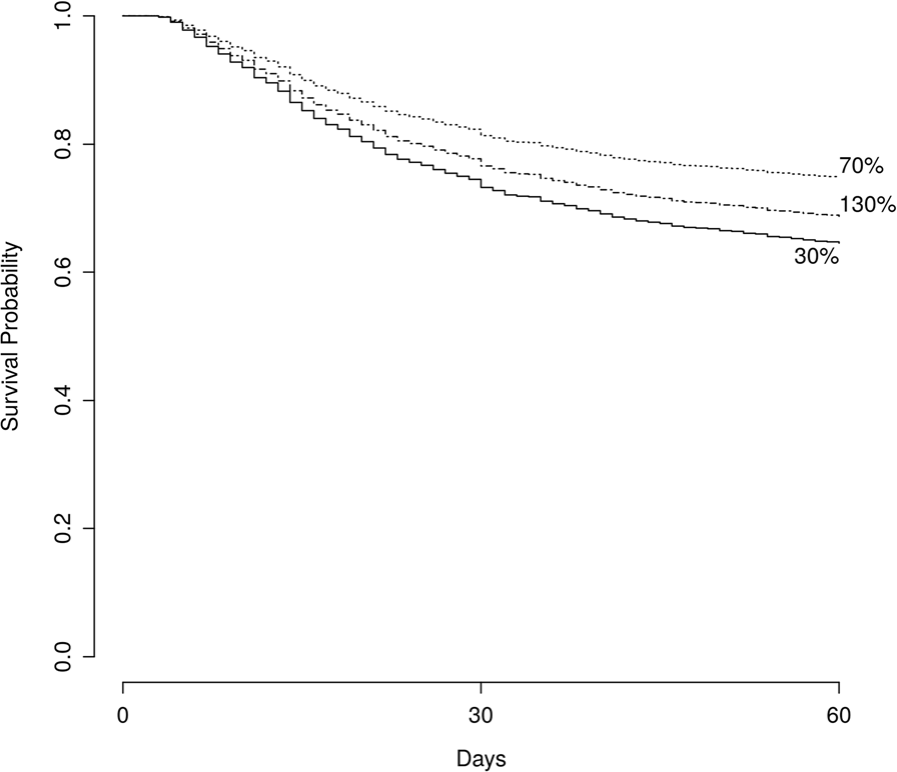
Association between administered calories/resting energy expenditure (Adcal/REE) percent and 60-day survival. *Labels* correspond to Adcal/REE percent

The association of protein intake, as a percent of requirement, with mortality by 60 days was also significant (HR 0.99, 95 % CI 0.98–0.99, *p* = 0.018). A survival curve based on the final model with specific AdCal/REE values is presented in Fig. 3. Substituting the AdCal/REE with daily total calories or REE alone was not significantly associated with mortality. The area under the curve (AUC) for the model was 0.75. After the validation process, the corrected AUC was 0.74, suggesting a stable and internally valid model.

Sensitivity analysis considering only measurements after the second day and adding evaluable nutrition days as a predictor did not change the HR, and the AdCal/REE remained significant (*p* = 0.0031), as did daily protein ingestion (*p* =0.01). Further restriction of the sample to patients who had >10 evaluable nutrition days (757 patients), and still controlling for evaluable nutrition days, produced similar results.

### Length of stay and length of ventilation

To assess the effect on LOS and LOV in univariate analysis, three groups based on the AdCal/REE were defined, namely <70 % (*n* = 110), 70–100 % (*n* = 390) and >100 % (*n* = 671). Median LOS in surviving patients was 12, 15 and 16.5 days for the three groups, respectively (*p* < 0.001 for the difference). Median days of ventilation in the three groups were 10, 13, and 14 days, respectively (*p* < 0.001).

## Discussion

In this retrospective observational study, which is to our knowledge the largest cohort studied using indirect calorimetry as opposed to predictive equations to determine energy requirements in mechanically ventilated, critically ill patients, we have demonstrated a non-linear, significant association between the percent AdCal/REE and mortality by 60 days. The results suggest that increasing the AdCal/REE to 70 % was associated with decreased mortality, while increases above that point, particularly as the curve increased >100 %, were associated with increasing mortality. In addition, protein ingestion was independently and significantly associated with decreased mortality. However, achieving calorie targets was associated with a longer ICU stay and length of ventilation.

Uncertainty about the optimal goals for nutritional support continues, fueled by conflicting results in the recent literature. Thus, an apparent lack of benefit with higher caloric intake was demonstrated by Arabi et al. [4] who compared permissive underfeeding to standard feeding (835 ± 297 vs 1299 ± 467 kcal/day, respectively) in patients who received the same amount of protein, and by Rice et al. in two studies comparing trophic to standard therapy (300 ± 149 vs 1418 ± 686 and 425 ± 141 vs 1385 ± 46 kcal/day, respectively) [5, 19]. On the other hand, a positive association between achieving caloric goals and outcome was demonstrated in observational studies [2, 3] and in RCTs by our group in the TICACOS study [1], and others that used indirect calorimetry [20, 21]. As suggested by Heyland et al., this discrepancy may be explained at least in part by the statistical methods used in the studies showing an apparent lack of effect, in particular the method accounting for duration of exposure to nutrition or length of stay. In this regard we have shown in our study an association between achieving 70 % of the AdCal/REE and improved survival, which remained consistent even when only measurements after the second day and adding evaluable nutrition days as a predictor were considered.

In contrast to many previous studies that used an arbitrary, predefined cutoff of caloric intake to define optimal nutrition, mainly based on predictive equations, we examined the AdCal/REE as a continuous variable which allowed us to assess the relationship between administered calories and mortality at various levels of intake. The resultant U-shaped curve revealed a decrease in mortality as the caloric intake was increased to 70 % of target calories, but this was followed by an increase in mortality, particularly as the curve increased >100 %.

Our findings are in accordance with those of Heyland et al. who used similar methodology to show that providing more than two-thirds of prescribed calories was associated with reduced mortality and suggested that providing >85 % of the caloric goal was associated with the best outcome [2]. In addition, they did not find an additional benefit of feeding >100 % of the target. However, the Heyland study used predictive equations to assess calorie goals, IC being used in only 0.5 % of their cases. By contrast, all our patients were assessed with IC, many of them having repeated measurements during the course of their stay so that their true and possibly changing metabolic needs may have been more accurately assessed. This difference in methods of assessing REE may have accounted for the difference in optimal targets noted in the two studies, namely 70 % vs. 85 %. In this regard, a recent position paper from the Multicentric Study Group for Indirect Calorimetry stated that IC is a tool of paramount importance necessary to optimize the nutrition therapy of patients with various pathological conditions, including critically ill patients [10]. It is important to note that due to a number of factors, there is typically a negative gap between administered and targeted calories; thus, calorie targets should be 100 % of REE.

In our study, increasing the delivered caloric intake >70 % of REE was associated with both a longer ICU stay and days of ventilation. These results confirm those found in the TICACOS study [1]. This might be explained by the fact that higher amounts of calories add to the work of breathing, thus, resulting in a longer time to wean from ventilation and thus, a longer ICU LOS. These complications were not evident in the Arabi and Rice studies in the groups receiving standard nutritional support [4, 5]. However, in both these studies, the standard therapy groups received lower calorie loads compared to our study, namely 1299 and 1300 kcal/day, respectively, compared to 1651 kcal/day. Complications were not mentioned in the Heyland study. This finding of more prolonged ventilation and ICU LOS could account for the U-shaped curve we noted where an optimal amount of calories may exist, while deviations from it, either above or below, may be associated with harm. The fact that caloric intake is not a binary variable, that the caloric goal is as yet unknown and that surrogate measures for IC that are often used are inaccurate, complicate the quest to find this optimal goal and might explain the contradicting results of past studies. If the effect of caloric intake on mortality is indeed U-shaped, it supports the more widespread use of accurately measured metabolic requirements, either by IC or by improved predictive equations.

Our study also supports the increasingly appreciated importance of protein in improving survival, as protein intake was linearly associated with decreased mortality in the multivariable model (HR 0.99, 95 % CI 0.98–0.99, *p* = 0.018) suggesting a 1 % reduction in mortality for every gram of daily protein ingested. These results echo other observational studies [22, 23] highlighting the importance of protein intake. In addition, it is worth noting that even with the use of supplemental parenteral nutrition, the realities of ICU make it difficult to achieve 100 % of caloric and protein targets.

Finally, it is interesting to note the reduced REE in patients who died, which has been suggested to be the result of multi-organ dysfunction in sepsis leading to metabolic shutdown [24]. This issue requires further elucidation. Again, this highlights the importance of IC- based REE measurements as metabolic needs may shift through the course of a critical illness.

Our study has several limitations. By the very nature of the observational design, inclusion of indirect calorimetry patients and the non-randomized administration of calories might introduce selection bias. As in these designs, especially in nutrition assessment trials, results need to be interpreted with caution as there is a risk that non-random allocation itself might influence the results, i.e. well-fed patients have a better prognosis irrespective of caloric requirements or that higher levels of caloric intake pre-select longer-surviving patients. In addition, while we have tried to account for confounders, we cannot rule out additional, as yet unknown ones.

Ours is a single-center study describing a unique critically-ill population, and the center-specific practices, which might limit the external validity. In addition the differences might not manifest in a short duration of stay. We have attempted to address these limitations in the design of our study as described in “Methods”. Thus, regarding selection and time bias, we restricted the sample size, excluded short-stay patients and used several sensitivity analyses that showed the stability of the results. The fact that the REE and the AdCal/REE percent were relatively stable in time >48 hours of hospitalization further supports a lack of time bias. In addition, our results show that “slightly underfed” patients, i.e. those receiving 70 % of target calories, fared better than optimally fed patients (those receiving 100 % of target calories), which argues against the notion that well-fed patients might have a better prognosis irrespective of nutrition demands. In addition we have tried to account for confounders, including disease severity.

## Conclusions

The findings of this study suggest that both underfeeding and overfeeding appear to be harmful to mechanically ventilated, critically ill patients. While an energy target of 100 % of assessed requirements remains the ideal goal, the reality of ICU inevitably precludes this ideal. In this regard we have shown that achieving an Adcal/REE of at least 70 % had a survival advantage, while a higher caloric intake, especially >100 % may be associated with harm. The optimal way to define caloric goals therefore requires an exact estimate, which is ideally performed using indirect calorimetry. These findings provide a basis for future randomized controlled trials comparing specific nutritional regimens based on indirect calorimetry measurements.

## Abbreviations

AdCal/REE: administered calories/resting energy expenditure
ANOVA: analysis of variance
APACHE: acute physiology and chronic health evaluation
AUC: area under the curve
HR: hazard ratio
IC: indirect calorimetry
ICU: intensive care unit
LOS: length of stay
LOV: length of ventilation
RCT: randomized controlled trial
SOFA: sequential organ failure assessment

## References

[1] Singer P, Anbar R, Cohen J, Shapiro H, Shalita-Chesner M, Lev S (2011). The tight calorie control study (TICACOS): a prospective, randomized, controlled pilot study of nutritional support in critically ill patients. Intensive Care Med.

[2] Heyland DK, Cahill N, Day AG (2011). Optimal amount of calories for critically ill patients: depends on how you slice the cake!. Crit Care Med.

[3] Alberda C, Gramlich L, Jones N, Jeejeebhoy K, Day AG, Dhaliwal R (2009). The relationship between nutritional intake and clinical outcomes in critically ill patients: results of an international multicenter observational study. Intensive Care Med.

[4] Arabi YM, Aldawood AS, Haddad SH, Al-Dorzi HM, Tamim HM, Jones G (2015). Permissive underfeeding or standard enteral feeding in critically ill adults. N Engl J Med.

[5] Rice TW, Wheeler AP, Thompson BT, Steingrub J, Hite RD, National Heart, Lung, and Blood Institute Acute Respiratory Distress Syndrome (ARDS) Clinical Trials Network (2012). Initial trophic vs full enteral feeding in patients with acute lung injury: the EDEN randomized trial. JAMA.

[6] Dvir D, Cohen J, Singer P (2006). Computerized energy balance and complications in critically ill patients: an observational study. Clin Nutr Edinb Scotl.

[7] Villet S, Chiolero RL, Bollmann MD, Revelly J-P, Cayeux RNM-C, Delarue J (2005). Negative impact of hypocaloric feeding and energy balance on clinical outcome in ICU patients. Clin Nutr Edinb Scotl.

[8] Reid CL (2007). Poor agreement between continuous measurements of energy expenditure and routinely used prediction equations in intensive care unit patients. Clin Nutr Edinb Scotl.

[9] Singer P, Berger MM, Van den Berghe G, Biolo G, Calder P, Forbes A (2009). ESPEN guidelines on parenteral nutrition: intensive care. Clin Nutr Edinb Scotl.

[10] Oshima T, Berger MM, De Waele E, Guttormsen AB, Heidegger C-P, Hiesmayr M, et al. Indirect calorimetry in nutritional therapy. A position paper by the ICALIC study group. Clin Nutr Edinb Scotl. 2016 10.1016/j.clnu.2016.06.010 27373497

[11] Taylor BE, McClave SA, Martindale RG, Warren MM, Johnson DR, Braunschweig C (2016). Guidelines for the provision and assessment of nutrition support therapy in the adult critically ill patient: Society of Critical Care Medicine (SCCM) and American Society for Parenteral and Enteral Nutrition (A.S.P.E.N.). Crit Care Med.

[12] Lev S, Cohen J, Singer P (2010). Indirect calorimetry measurements in the ventilated critically ill patient: facts and controversies–the heat is on. Crit Care Clin.

[13] Weir JBDB (1949). New methods for calculating metabolic rate with special reference to protein metabolism. J Physiol.

[14] Harrell F. Regression modeling strategies: with applications to linear models, logistic and ordinal regression, and survival analysis. Springer; 2015

[15] R Core Team (2015). R: A Language and Environment for Statistical Computing.

[16] Dowle M, Srinivasan A, Short T, Saporta SL, with contributions from R, Antonyan E. data.table: Extension of Data.frame. 2015. Available from: http://CRAN.R-project.org/package=data.table

[17] Wickham H (2009). ggplot2: Elegant Graphics for Data Analysis.

[18] Harrel FEJ (2016). rms: Regression Modeling Strategies.

[19] Rice TW, Mogan S, Hays MA, Bernard GR, Jensen GL, Wheeler AP (2011). Randomized trial of initial trophic versus full-energy enteral nutrition in mechanically ventilated patients with acute respiratory failure. Crit Care Med.

[20] Petros S, Horbach M, Seidel F, Weidhase L (2016). Hypocaloric vs normocaloric nutrition in critically ill patients: a prospective randomized pilot trial. JPEN J Parenter Enteral Nutr.

[21] Heidegger CP, Berger MM, Graf S, Zingg W, Darmon P, Costanza MC (2013). Optimisation of energy provision with supplemental parenteral nutrition in critically ill patients: a randomised controlled clinical trial. Lancet Lond Engl.

[22] Weijs PJM, Stapel SN, de Groot SDW, Driessen RH, de Jong E, Girbes ARJ (2012). Optimal protein and energy nutrition decreases mortality in mechanically ventilated, critically ill patients: a prospective observational cohort study. JPEN J Parenter Enteral Nutr.

[23] Nicolo M, Heyland DK, Chittams J, Sammarco T, Compher C (2016). Clinical outcomes related to protein delivery in a critically ill population: a multicenter, multinational observation study. JPEN J Parenter Enteral Nutr.

[24] Singer M (2007). Mitochondrial function in sepsis: acute phase versus multiple organ failure. Crit Care Med.

